# Genetic Architecture of Myopia and Its Implications for Risk Stratification and Prognosis

**DOI:** 10.3390/diagnostics16040536

**Published:** 2026-02-11

**Authors:** Yuri Seo, Dongheon Surl, Jinu Han

**Affiliations:** 1 Institute of Vision Research, Department of Ophthalmology, Gangnam Severance Hospital, Yonsei University College of Medicine, Seoul 06273, Republic of Korea; yrseo@yuhs.ac; 2 Institute of Vision Research, Department of Ophthalmology, Yongin Severance Hospital, Yonsei University College of Medicine, Yongin 16995, Republic of Korea; 3 Institute of Vision Research, Department of Ophthalmology, Severance Hospital, Yonsei University College of Medicine, Seoul 03722, Republic of Korea

**Keywords:** myopia, genetics, genome-wide association study, polygenic risk score, risk stratification, prognosis, early-onset high myopia

## Abstract

Myopia is a prevalent ocular condition with marked heterogeneity in onset and progression. Although diagnosis is straightforward, predicting disease trajectories and identifying risks of high or pathologic myopia remain main clinical challenges. Advances in human genetics have substantially reshaped current understanding of myopia, revealing a complex architecture involving common polygenic susceptibility, rare high-impact variants, and cumulative genetic risk burden. Large-scale genome-wide association studies demonstrate that myopia-related variants are enriched in regulatory and signaling pathways that modulate retinal neuronal and glial responses to visual and metabolic stimuli, while exome sequencing studies highlight overlap between early-onset high myopia and inherited retinal or syndromic disorders. Polygenic risk scores further translate common-variant burden into quantitative measures of genetic susceptibility, enabling population-level risk stratification and early risk assessment, albeit with performance differences across ancestries and clinical outcomes. Together, these findings delineate a multilayered genetic framework for myopia and support the role of genetic information as a complementary component of prognostic assessment. Integration of genetic data with longitudinal clinical and environmental information may further improve the prediction of myopia trajectories and facilitate more individualized management strategies.

## 1. Introduction

Myopia is one of the most prevalent ocular conditions worldwide, with particularly high prevalence in East Asian populations [1]. In clinical practice, myopia is readily diagnosed using standard refractive assessment and ocular biometry. Despite this diagnostic simplicity, myopia exhibits marked clinical heterogeneity in terms of age at onset, rate of progression, and long-term outcomes [1,2,3]. While many individuals maintain stable, low grade myopia, others experience rapid axial elongation that progresses to high or pathologic myopia, leading to vision-threatening complications such as myopic maculopathy, retinal detachment, and glaucoma [2,4]. Consequently, the principal challenge in myopia management lies not in diagnosis but in predicting disease course and long-term risk.

**Figure 1:**
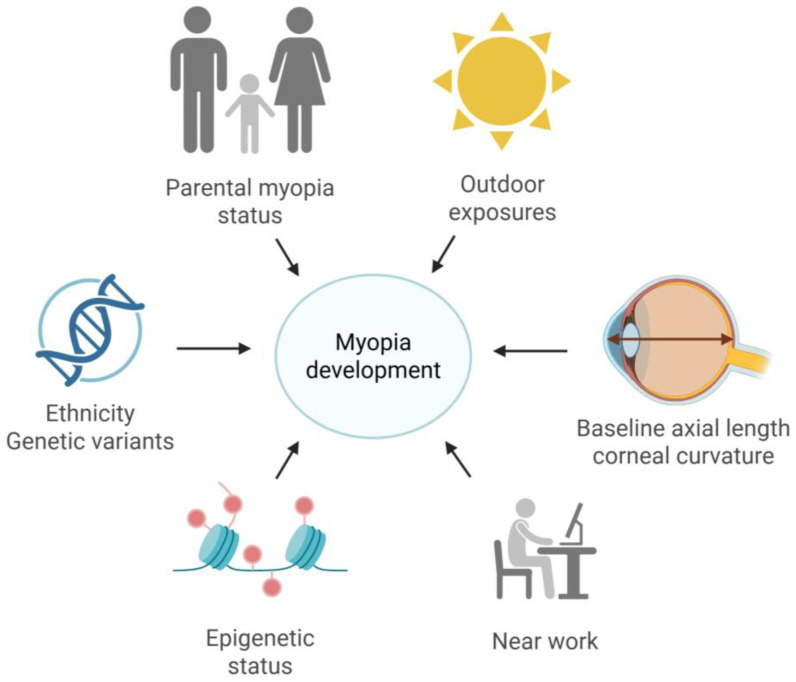
Multifactorial contributors to myopia development. Myopia arises from the interaction of genetic susceptibility, environmental exposures, ocular biometric factors, and epigenetic influences, contributing to substantial interindividual variability in disease onset and progression. Created with https://www.Biorender.com.

Myopia development is a complex, multifactorial process influenced by the interplay of genetic predisposition, environmental exposures, and ocular biometric factors. Established contributors include parental myopia, nearwork and outdoor exposure, baseline ocular anatomy such as axial length and corneal curvature, as well as broader genetic and epigenetic influences related to ethnicity and developmental context (Figure 1). This multifactorial architecture underlies the substantial heterogeneity observed in myopia onset and progression, limiting the predictive value of isolated clinical measurements.

Baseline clinical measures, including refraction and axial length assessed at a single time point, provide limited insight into individual trajectories of progression or future complications. Genetic factors offer complementary information on intrinsic susceptibility to myopia development and progression, helping to explain interindividual variability that is not captured by conventional clinical phenotyping.

The purpose of this review is to summarize recent advances in myopia genetics and to discuss how genetic information can complement standard clinical assessment in predicting myopia progression and long-term outcomes. We integrate evidence from genome-wide association studies (GWASs), exome sequencing (ES) studies, rare variant analyses, and gene–environment interaction studies to highlight the evolving role of genetics in myopia risk stratification and prognostic evaluation. This genetic perspective is considered in the context of prior longitudinal studies demonstrating that baseline clinical measures, such as spherical refractive error, can predict myopia onset [5,6].

## 2. Initial Locus Discovery and Genome-Wide Association Studies in Myopia

Early family and twin studies established that refractive error is highly heritable [7,8], yet early candidate gene approaches were limited by poor reproducibility and incomplete genomic coverage. Initial GWASs of myopia were therefore conducted across several population-based cohorts, including the Rotterdam Study, ALSPAC, GERA, and multiple East Asian cohorts (Table 1) [9,10,11]. These early efforts provided proof-of-concept for common-variant susceptibility but were constrained by modest sample sizes and limited cross-cohort replication. The field advanced decisively with large-scale meta-analyses led by the Consortium for Refractive Error and Myopia (CREAM), established in 2011 to harmonize phenotypes and integrate genetic data across international cohorts [12,13].

CREAM’s early work strengthened evidence for common-variant contributions to myopia by independently validating the previous reported association at the 15q14 locus harboring *GJD2*, which encodes connexin-36 and plays a key role in retinal signal transmission through gap junctions [12,14]. Building on this foundation, a landmark CREAM GWAS meta-analysis identified 24 novel loci for refractive error and replicated many loci independently detected by 23andMe using a pragmatic phenotype—age of onset of spectacle wear—demonstrating that large sample size and careful phenotypic harmonization can yield consistent genetic signals across distinct study designs [12,15].

As CREAM expanded, the scale and resolution of locus discovery increased substantially. A subsequent meta-analysis including 160,420 participants, with 95,505 individuals in independent replication cohorts, expanded the number of validated loci to 161, firmly establishing the highly polygenic architecture of refractive error [14]. Importantly for ophthalmic interpretation, these loci were not restricted to genes related to scleral structure or ocular size. Instead, pathway analyses highlighted biological processes such as cell–cell adhesion, synaptic transmission, calcium and cation channel activity, and growth-related signaling pathways including MAPK and TGF-β/SMAD, alongside genes involved in retinal physiology and light processing (e.g., *RGR*, *RP1L1*, *RORB*, and *GNB3*) and dopamine-related signaling (*DRD1*) [14]. Collectively, these findings support a model in which altered retinal and neuronal signaling acts upstream of axial elongation, rather than myopia being purely a disorder of scleral biomechanics.

In the largest CREAM-led GWAS meta-analysis to date, Hysi and colleagues identified 336 novel loci associated with refractive error [16]. A meta-analysis of GWAS indicated that genetic variants related to eye structure, retinal light transmission, circadian rhythms, and pigmentation are associated with refractive errors. Collectively, these variants account for 18.4% of heritability and contribute to improving population-level risk stratification for myopia (Table 1) [16].

Although individual GWAS variants exert only small effects, aggregation of common risk alleles can yield clinically meaningful discrimination at the population level. Early polygenic analyses demonstrated up to an approximately 40-fold difference in myopia risk between individuals in the extreme deciles of a polygenic risk score derived from genome-wide significant variants [14]. These findings indicate that common-variant genetics captures important information on susceptibility and provides a foundation for population-based risk stratification.

At the same time, GWASs have clarified the current scope and limitations of genetic information in clinical practice. Although myopia is highly heritable, currently identified common variants explain a substantial but incomplete proportion of this heritability, with recent polygenic scores accounting for up to approximately 20% of the variance in refractive error [17,18]. Despite this progress, a substantial proportion of heritable variation remains unexplained, reflecting the well-recognized concept of missing heritability. This residual heritability may arise from contributions of rare variants with larger effect sizes, structural variation, gene–gene (GxG) and gene–environment (GxE) interactions, as well as phenotypic heterogeneity, which are not fully captured by conventional GWASs. As a result, common-variant associations alone are insufficient as standalone tools for individualized clinical decision making, but provide valuable insight into genetic susceptibility when interpreted in conjunction with clinical and environmental factors.

These limitations have prompted complementary approaches to more fully capture the genetic architecture of myopia. Rare-variant analyses based on ES and genome sequencing (GS) have sought to identify variants with larger effect sizes, particularly in early-onset and severe forms of myopia. In parallel, the aggregation of common variants through polygenic risk scoring has emerged as a strategy to translate GWAS findings into quantitative estimates of genetic risk. Together, these approaches represent critical next steps toward advancing myopia genetics from population-level association to individualized prognostic stratification and clinically meaningful risk prediction.

**Table 1:** Genome-wide association studies identifying genetic loci associated with myopia and related ocular traits.

Study	Ancestry	Sample Size	Key Contribution
Nakanishi et al. (2009) [9]	Japanese	830 cases/1911 controls	First GWAS to identify a susceptibility locus for pathological myopia in an East Asian population.
Hysi et al. (2010) [10]	European	17,684	Identified a susceptibility locus for refractive error at 15q25 (*RASGRF1*), highlighting the role of retinal neuronal signaling in myopia.
Solouki et al. (2010) [11]	European	15,608	Identified a common susceptibility locus at 15q14 for refractive error.
Verhoeven et al. (2013, CREAM) [12]	Multi-ancestry	45,758	First large CREAM meta-analysis identifying 24 novel loci for refractive error.
Kiefer et al. (2013) [15]	European	54,094	Largest GWAS using age of myopia onset as the phenotype.
Tedja et al. (2018) [14]	Multi-ancestry	160,420	Large-scale GWAS meta-analysis highlighting light-induced retinal signaling pathways and extracellular matrix remodeling in myopia.
Hysi et al. (2020) [16]	European	542,934	Large-scale GWAS meta-analysis for refractive error, identifying 336 loci and demonstrating enrichment of genetic risk in retinal neuronal and glial cell types.
Plotnikov et al. (2021) [19]	European	22,180	Demonstrated that genetic determinants of normal eye size differ from those of refractive error and myopia.
Jiang et al. (2023) [20]	Multi-ancestry	19,420	Identified axial length associated loci with sharing genetic architecture with myopia.

## 3. Link to Inherited Retinal Diseases and Syndromic Diseases

It is well established that early-onset high myopia (eoHM) is frequently associated with early-onset hereditary retinal diseases, including achromatopsia, congenital stationary night blindness (CSNB), early-onset severe retinal dystrophy (EOSRD), Leber congenital amaurosis (LCA), and familial exudative vitreoretinopathy (FEVR) (Figure 1) [21]. Consequently, comprehensive imaging evaluations—such as optical coherence tomography (OCT) and fundus autofluorescence (FAF)—are essential in the clinical assessment of these patients, with electroretinography (ERG) performed when indicated. The IMI report provides detailed guidelines on the diagnostic approach for infants and children with high myopia (HM), including which tests to consider and when [22].

In patients with CSNB, specifically, fundus photography, OCT, and FAF often reveal no structural abnormalities. Therefore, if a patient presents with high myopia (HM) and reduced visual acuity disproportionate to age-normative values, it is crucial to conduct ERG and genetic testing. Furthermore, given that nystagmus is associated with approximately 50–70% of CSNB cases, the presence of infantile nystagmus warrants further diagnostic investigation, even in the presence of a normal-appearing retina. Additionally, as optic atrophy is frequently observed in *CACNA1F*-related CSNB, clinical suspicion should remain high if retinal nerve fiber layer (RNFL) thinning is detected on RNFL mapping in conjunction with significant refractive errors [23]. Finally, since HM is a common feature in syndromic conditions such as Stickler syndrome, Knobloch syndrome, and Marfan syndrome, a thorough medical history is imperative [24]. Clinicians must carefully evaluate for systemic manifestations—including Pierre Robin sequence, skeletal abnormalities, chest deformities, heart valvular diseases, and lens dislocation—to identify any syndromic features beyond HM (Table 2). Furthermore, given that HM is frequently associated with various large-scale chromosomal abnormalities such as deletions and duplications, it is crucial to perform deep phenotyping and molecular characterization to pursue etiologies and appropriate managements [25].

## 4. Exome Sequencing in HM or eoHM

While common myopia is often attributed to environmental factors like intensive nearwork, eoHM appearing before school age is driven predominantly by genetic predispositions [22,26]. Recent advancements in ES have allowed for the identification of numerous rare variants and novel genes that clarify the biological mechanisms of axial elongation [27,28]. A significant finding across multiple ES cohorts is the high prevalence of mutations in genes associated with inherited retinal diseases (IRDs), collectively known as RetNet genes, in eoHM patients [26,29]. Potential pathogenic variants in these genes are detected in approximately 23.4% to 23.8% of eoHM probands, a rate significantly higher than in late-onset cases [29]. The diagnostic yield of ES and GS in eoHM cases is much lower than for IRDs. One potential explanation for this result is that many cases of eoHM may be polygenic rather than monogenic [26,29,30]. Frequently implicated genes include *COL2A1*, *COL11A1*, *RPGR*, and *CACNA1F*. These findings suggest that some cases of eoHM are subclinical or early manifestations of syndromes, like Stickler syndrome, or various inherited retinal dystrophies. ES has been instrumental in discovering genes that regulate the structural integrity of the sclera and the metabolism of the extracellular matrix (ECM). The *ZNF644* gene was identified as a cause of monogenic HM through its role as a transcription factor in eye development [31]. Similarly, variants in *SLC39A5* have been linked to axial elongation through interference with the BMP/TGF-*β* signaling pathway [32]. Recent research identified *KDELR3* as a significant risk gene; its protein-truncating variants lead to thinned scleral and choroidal layers [27]. Other important structural genes identified include *LOXL3*, where null mutations cause autosomal recessive eoHM [28], and *P4HA2*, which affects collagen hydroxylation [33].

Beyond structural components, ES highlights the importance of visual signaling. The *GLRA2* gene, encoding a glycine-gated chloride channel subunit, has been shown to cause HM by impairing visual transmission [34]. Mutations in *ARR3* follow a unique X-linked female-limited inheritance pattern and represent a common cause of Mendelian eoHM [35]. Furthermore, sex-stratified exome-wide association studies (ExWAS) identified *CHRNB1* as a male-specific gene, with deficiency leading to mitochondrial dysregulation [36]. Other candidates like *PPEF2* and *PSMD3* impact retinal pigment epithelium (RPE) function and cell apoptosis [37,38]. Table 3 summarizes the associated genes identified by ES and provides an overview of the relevant studies.

## 5. Polygenic Risk Scoring

Polygenic risk scores (PRSs) have emerged as a strategy to translate GWAS findings into quantitative estimates of genetic susceptibility by aggregating the effects of numerous common variants across the genome [46,47]. Given the highly polygenic architecture of refractive error, PRSs leverage information from thousands or millions of variants, each conferring small effects, to model cumulative genetic risk beyond what can be inferred from individual loci [46].

Across multiple population-based studies, PRSs have consistently demonstrated the ability to stratify myopia risk at the population level [46,47]. Individuals in the highest-polygenic-risk groups are several times more likely to develop myopia than those in the lower-risk groups, particularly when comparing the extreme ends of the PRS distribution [46]. These findings indicate that common-variant genetic burden captures meaningful information on myopia susceptibility and can distinguish high-risk groups even before clinical manifestation [46,47].

However, the performance of PRS is highly dependent on ancestry and phenotype definition. Most PRS models have been derived from GWASs conducted predominantly in populations of European ancestry, and their predictive accuracy decreases when applied to East Asian or other non-European cohorts [48,49]. Studies in Chinese and multi-ethnic Asian populations have demonstrated attenuated effect sizes and reduced discriminatory power, reflecting differences in linkage disequilibrium structure, allele frequencies, and environmental exposures such as educational intensity and nearwork behavior [48,49]. These observations highlight ancestry mismatch as a major barrier to the universal application of current PRS models and emphasize the need for ancestry-specific or multi-ancestry discovery datasets [47,48].

The clinical relevance of PRSs also varies by outcome. In pediatric cohorts, PRSs show stronger association with myopia onset or presence, and selected studies have also demonstrated significant associations with myopia progression and axial elongation [47,50]. Children with higher PRS are more likely not only to develop myopia but also experience faster refractive progression [50]. However, the magnitude of this effect is moderate, explaining a limited proportion of variance in longitudinal refractive change, and current evidence is largely derived from ancestry-specific cohorts, including pediatric studies conducted in children of Chinese ancestry [47,50]. These findings suggest that while genetic susceptibility contributes to progression risk, longitudinal myopia progression is additionally shaped by age-dependent biological process and environmental modifiers that are not fully captured by current PRS models [47].

Similarly, although PRSs for refractive error are associated with an increased risk of HM, its utility in predicting myopia-related degenerative complications remains uncertain [17,48]. Studies examining outcomes such as myopic macular degeneration have reported heterogeneous findings, with most evidence suggesting that observed associations are largely mediated by refractive severity and show little independent predictive value after adjustment [17,48]. These findings imply that structural and degenerative sequelae of myopia involve additional genetic and non-genetic mechanisms distinct from those governing refractive error susceptibility [17,47].

Taken together, current evidence supports PRS as a population-level screening and risk awareness tool rather than a standalone clinical test or an individual decision-making instrument. Its greatest value lies in early risk stratification, particularly before conventional clinical measures such as cycloplegic refraction or axial length begin to reflect myopia risk. In this context, PRS may help identify subgroups of children who could benefit from closer monitoring and preventive counseling, rather than guiding treatment selection or predicting individual clinical outcomes. Based on currently available evidence, PRS does not yet support personalized therapeutic decision-making or the independent prediction of myopia-related degenerative complications.

## 6. Gene–Environment (GxE) Interaction in Myopia Development

It is generally believed that myopia results from the interaction of genes and environment [51]; neither genetic nor environmental factors alone can fully explain the etiology of myopia. According to the GWAS meta-analysis by Tedja et al. (2018), common genetic variants identified in large-scale genomic studies explain approximately 12% of the variance in refractive error [14]. In contrast, traditional twin studies have reported that refractive error is highly heritable, with genetic factors estimated to account for approximately 60–80% [7,52]. This suggests that environmental factors play a significant role in the remaining variance. Therefore, understanding GxE interactions is a crucial element for monitoring the development and treatment of myopia; however, research in this area remains insufficient. This GxE interaction highlights the necessity of considering the complex interplay between genetic predispositions—such as those related to an individual’s outdoor exposure or scleral remodeling—and actual environmental exposure variables. Specifically, the onset and progression of myopia are explained by the interaction between genetic risk alleles (e.g., *AREG*, *VIPR2* [rs2071623]) and environmental risk factors, including nearwork time, education level, and reading duration [53]. A major limitation in GxE research has been the reliance on questionnaires to assess environmental factors like nearwork and outdoor time. This traditional method is subject to significant recall bias and social desirability bias, with studies showing that self-reported data tends to overestimate both nearwork duration and outdoor exposure. This lack of precision can weaken the statistical power to detect genuine GxE interactions. Recently, wearable devices such as the Clouclip (a spectacle-mounted sensor) and smartwatches have enabled the objective measurement of environmental factors by tracking outdoor exposure and continuously monitoring light intensity [53]. These advancements are expected to enhance the accuracy and reliability of environmental factors affecting myopia progression.

## 7. Clinical Application: Myopia Progression Modeling and Pharmacogenetics

While genetic architecture of myopia susceptibility is well documented involving variants in various genes such as *ZFHX1B*, *SNTB1*, and *GJD2* [49,50,54], research into the pharmacogenetics of myopia control is still in its early stages. Currently, non-selective muscarinic antagonists (e.g., atropine) are commonly used medication in controlling myopia progression [55]. However, clinical data reveals significant heterogeneity in treatment response. It is hypothesized that genetic variations in *CHRM1*, *CHRM2*, and *CHRM3* genes might influence muscarinic receptor binding affinity or density in the retina or sclera. Polymorphism in these receptor genes could explain resistance to low-dose atropine eye drops. In addition, genes involved in scleral remodeling pathway (e.g., *MMP2* and *TIMP2*) have been proposed as potential pharmacogenetic markers [56,57]. Theoretically, genetic variants in downstream collagen synthesis, endoplasmic reticulum stress of the sclera (e.g., *EIF2AK3*, *ATF6*), or metalloprotease or serine protease pathway could impact the drug’s ability to slow axial elongation [58,59].

Future studies may determine whether pharmacogenomic screening could aid in understanding individual variations in drug response by assessing markers in muscarinic receptors and downstream signaling pathways. Furthermore, genetic insights could potentially inform drug discovery for myopia-associated pathways. Investigating the pharmacogenetic profile of myopia remains an area for future research, with the ultimate goal of optimizing childhood treatment strategies.

## 8. Gene Loci and Possible Mechanisms in the Eye

**Figure 2:**
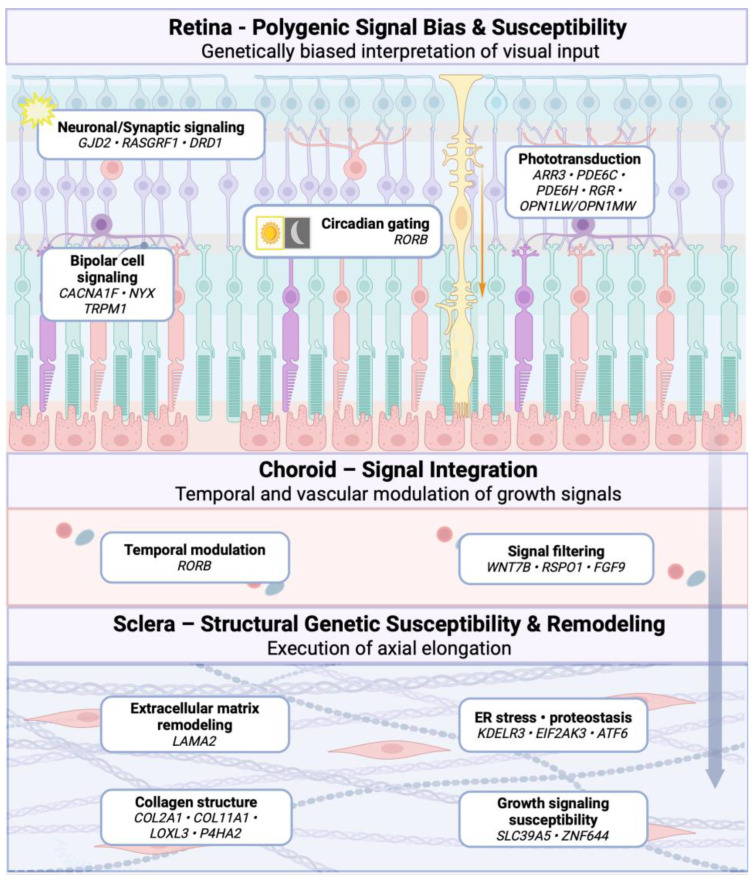
Genetic architecture and tissue-specific mechanisms in myopia. This schematic summarizes a conceptual integrated retina–choroid–sclera model of myopia based on genetic susceptibility and integrative post-GWAS analyses of myopia-associated loci. In the retina, polygenic risk is enriched in neuronal and glial signaling pathways, including synaptic transmission (*GJD2*, *RASGRF1*, *DRD1*), phototransduction (*ARR3*, *PDE6C*, *PDE6H*, *RGR*, *OPN1LW/OPN1MW*), bipolar cell signaling (*CACNA1F*, *NYX*, *TRPM1*), and circadian regulation (*RORB*), which are associated with differences in how visual input may be sensed and temporally gated rather than directly determining eye size. The choroid acts as a dynamic signal integration layer, where circadian and Wnt-related pathways (*RORB*, *WNT7B*, *FGF9*) are proposed to influence the timing and transmission of growth-related signals. In the sclera, genetically susceptible structural and stress-responsive pathways involving extracellular matrix remodeling (*LAMA2*, *COL2A1*, *LOXL3*), endoplasmic reticulum stress and proteostasis (*KDELR3*, *EIF2AK3*, *ATF6*), and growth-related signaling (*SLC39A5*, *ZNF644*) are implicated in biomechanical remodeling processes and axial elongation. Together, myopia-associated genetic variants are proposed to contribute to ocular growth through coordinated, tissue-specific modulation of signaling and structural response. Created with https://www.Biorender.com.

GWAS have identified numerous loci associated with refractive error and myopia; however, these variants individually confer small effect sizes and rarely map to a single dominant biological pathway. Post-GWAS functional annotation consistently indicates that myopia-associated genetic risk is enriched in regulatory gene networks rather than structural components, suggesting that genetic susceptibility may preferentially be associated with regulatory and signaling pathways that are associated with tissue-specific biological responses. Accumulating experimental evidence is consistent with a model in which myopia-associated loci are linked to context-dependent metabolic, circadian, and stress-responsive processes across the retina, choroid, and sclera, with axial elongation conceptualized as an integrated outcome rather than a linear genetic program. An integrated retina–choroid–sclera model linking genetic susceptibility to axial elongation is schematically illustrated in Figure 2.

Myopia-associated loci show preferential enrichment in genes expressed in retinal neuronal and glial populations, supporting the retina as the key site where genetic susceptibility may interact with visual input [60]. Proteomic and transcriptomic studies identify Müller glial cells as key intermediaries, undergoing early metabolic reprogramming characterized by enhanced glycolysis, redox imbalance, and hypoxia-related signaling during myopia development [61,62]. These findings suggest that genetic variation may influence the sensitivity of retinal cells to visual and metabolic stress, rather than encoding direct growth signals, thereby potentially affecting the intensity and duration of signaling pathways associated with myopia development [60,62,63].

The functional impact of genetically primed retinal pathways is strongly influenced by circadian timing. Diurnal transcriptomic analyses demonstrate that many myopia-related genes, including those overlapping with GWAS loci, exhibit differential expression only during specific phase of the light-dark cycle, particularly the light phase [64]. This temporal gating may offer a mechanistic framework to help explain variability across experimental studies and highlights circadian biology as a critical modifier of GxE interaction in myopia [64,65].

As illustrated in Figure 2, signals initiated in the retina are thought to be subsequently transmitted through the choroid, where their timing and intensity are actively modulated, rather than being passively conveyed to the sclera. Although relatively few myopia-associated loci show direct choroidal specificity, choroid exhibits sustained, dark phase-dominant gene expression changes that integrate circadian, vascular, and metabolic cues before growth-related signals reach the sclera [60,64,66].

Despite limited direct enrichment of myopia-associated loci in sclera structural genes, axial elongation ultimately reflects scleral remodeling mediated by metabolic and cellular stress pathways. Experimental myopia is associated with increased glycolytic activity in scleral fibroblasts, leading to lactate accumulation, epigenetic regulation via histone acetylation, and activation of transcriptional programs, such as Notch signaling, that promote extracellular matrix remodeling [67]. In parallel, scleral endoplasmic reticulum stress mediated by the PERK-ATF6 axis has been shown to be necessary for myopic axial elongation, suggesting a potential functional link between regulatory genetic variation and biomechanical change in the eye [58].

Beyond DNA sequence variation, epigenetic mechanisms further integrate genetic susceptibility with environmental exposure. Targeted methylation analyses demonstrate subtle, locus-specific methylation changes in immediate early genes such as *EGR1*, occurring within restricted retinal cell populations rather than globally [68]. Together, their findings support an integrated retina–choroid–sclera model in which myopia-associated gene loci may contribute to tissue-specific responsiveness to visual input, metabolic stress, and circadian timing, defining a permissive biological landscape upon which environmental and temporal factors determine whether susceptibility is translated into pathological axial elongation [60,63,65].

## 9. Conclusions

Despite extensive research into the genetics of myopia, genetic variants alone do not fully account for the onset and progression of the disease. Furthermore, the functional mechanisms underlying many non-coding variants identified via GWASs remain elusive [47]. Given that myopia is a multifactorial condition driven by complex interactions among genetics, environment, and lifestyle, a comprehensive analytical approach that integrates these diverse determinants is essential. Moreover, current short-read sequencing technologies face limitations in accurately resolving complex genomic regions, such as the *OPN1LW*/*OPN1MW* gene arrays [69]. Therefore, in the near future, the convergence of objective lifestyle monitoring via smart wearables (e.g., smart glasses and watches) and advanced genomic insights from long-read sequencing—specifically for CNV analysis—is expected to revolutionize the field. These advancements will likely facilitate more accurate disease modeling and pharmacogenomics, ultimately paving the way for the development of novel therapeutic agents and personalized treatment strategies. We anticipate that if future studies elucidate the precise mechanisms of myopia development and provide information on individual risks, personalized counseling and risk assessment will become possible.

## Figures and Tables

**Table 2:** Inherited retinal diseases associated with high myopia.

Condition	Genes	Fundus Features	Syndromic Features	Characteristics
Achromatopsia	*PDE6C*, *PDE6H*, *GNAT2*	Normal-looking fundus in early childhood	None	Infantile nystagmus with fine amplitude was noted. Other genes associated with achromatopsia, such as *CNGA3* and *CNGB3*, are classically associated with HM.
Blue cone monochromacy	*OPN1MW*, *OPN1LW*	Normal-looking fundus in early childhood	None	Infantile nystagmus and color blindness were noted in nearly all patients.
CSNB	*CABP4*, *CACNA1F*, *GPR179*, *GRM6*, *LRIT3*, *NYX*, *TRPM1*	Normal	None	The X-linked genes in CSNB (*CACNA1F* and *NYX*) and autosomal recessive *TRPM1* are the most common genes associated with HM.
Leber congenital amaurosis	*CRB1*, *GUCY2D*, *TULP1*	Granular retinal dystrophy, nummular retinal degeneration	None	Most of genes associated with LCA cause high hyperopia, but HM is frequently associated with *TULP1*.
Early onset severe retinal dystrophy	*CFAP418*, *FAM161A*, *IFT140*, *RBP3*, *RPGR*, *RP2*	Salt and pepper retinopathy	Usually none	X-linked RP genes, such as *RPGR* and *RP2*, usually cause HM in childhood. Polydactyly and obesity were reported in *CFAP418*. Renal failure or skeletal anomalies (Mainzer–Saldino syndrome) can be associated with *IFT140*.
FEVR	*LRP5*, *FZD4*, *TSPAN12*, …	Retinal temporal dragging	Osteoporosis	Biallelic variants in *LRP5* cause osteoporosis-pseudoglioma.
Stickler syndrome	*COL2A1*, *COL11A1*, …	Vitreous degeneration, retinal detachment, cataract	Pierre Robin sequence, midface hypoplasia, sensorineural hearing loss	Membranous vitreous in *COL2A1*, beaded vitreous in *COL11A1*. Prophylactic cryotherapy should be done to prevent retinal detachment in *COL2A1*-related vitreoretinopathy. Sensorineural hearing loss is more severe in *COL11A1*.
Marfan syndrome	*FBN1*	Ectopia lentis, flattened cornea, poorly dilated pupil	Chest deformity, cardiac valve abnormalities, dural ectasia	Cardiac echo and regular examination are needed.
Knobloch syndrome	*COL18A1*	Vitreoretinal degeneration, collapsed/syneretic vitreous	Occipital skull abnormalities	Brain MRI is necessary to find associated brain anomalies including encephalocele.
Cohen syndrome	*VPS13B*	Bull’s eye maculopathy, retinal dystrophy	Microcephaly, hypotonia, tapering finger, truncal obesity	Frequent infections may occur due to neutropenia.
Poretti-Boltshauser syndrome	*LAMA1*	Retinal dystrophy	Cerebellar cysts and dysplasia, developmental delay, and intellectual disability	Nystagmus and oculomotor apraxia have been reported.
*ADAMTS18*-related ocular disease	*ADAMTS18*	Retinal dystrophy, anterior segment dysgenesis, microcornea, thick lens diameter	None	Abnormal facial features were reported only in one family.

CSNB = congenital stationary night blindness; FEVR = familial exudative vitreoretinopathy; HM = high myopia; MRI = magnetic resonance imaging.

**Table 3:** Exome sequencing studies in high myopia.

Study/Citation	Causative/Candidate Gene(s)	Mode of Inheritance	Sequencing Method	Number of Participants	Key Findings
Shi et al. (2011) [31]	*ZNF644*	AD	ES	1 family/300 sporadic cases	Identified *ZNF644* as a causative gene for monogenic HM via axial elongation.
Zhao et al. (2013) [39]	*PRIMPOL*	AD	ES	1 family/270 sporadic cases	*PRIMPOL* identified as a susceptibility gene; mutations correlate with very high refractive errors.
Li et al. (2016) [28]	*LOXL3*	AR	ES	298 eoHM probands	Found homozygous/compound heterozygous null mutations in *LOXL3* as a cause for AR-eoHM.
Zhou et al. (2018) [29]	*COL2A1*, *COL11A1*, *RPGR*, *CACNA1F*	AD, AR, XL	ES	325 eoHM/195 loHM probands	23.4% cases of eoHM involve RetNet mutations; eoHM is genetically distinct from loHM.
Napolitano et al. (2018) [33]	*P4HA2*	AD	ES	1 family (7 members)	Novel *P4HA2* missense variant causes defective collagen hydroxylation and axial elongation.
Pan et al. (2019) [40]	*TNFRSF21*	AD	ES	1 family/220 sporadic cases	*TNFRSF21* variants significantly increase RPE cell apoptotic levels in non-syndromic HM.
Swierkowska et al. (2021) [41]	*FLRT3*, *SLC35E2B*	Likely AD	ES	17 people (7 families)	Rare variants in *FLRT3* and *SLC35E2B* may contribute to HM in Central European families.
Liu et al. (2021) [42]	*CSMD1*, *PARP8*, *ADAMTSL1*, *FNDC3B*	AD/Heterozygous	ES	27 HM families	Identified four novel candidate genes in Northwest China population.
Yang et al. (2023) [32]	*PRIMPOL*, *SLC39A5*, *P4HA2*, *CPSF1*	AD, AR, XL	Familial ES	30 families	Found IRD-associated genes in 76.67% of families; expanded eoHM mutation spectrum.
Tian et al. (2023) [34]	*GLRA2*	XL	ES & Linkage	2 families/200 sporadic cases	*GLRA2* variants impair photoperception and visual transmission, leading to axial elongation.
Chen et al. (2023) [38]	*PSMD3*	AD	ES	1 family/179 sporadic cases	*PSMD3* mutations cause RPE dysfunction and significant axial length elongation.
Wang et al. (2023) [43]	*OPN1LW* (Haplotypes)	XL	ES/TES	1226 families/9304 controls	LVAVA/LIVVA cause eoHM alone; LIAVA/truncations cause eoHM with protanopia.
Jing & Yi (2024) [37]	*PPEF2*	AD	ES and amplicon-seq	1 family/100 sporadic cases	Identified *PPEF2* as a novel HM gene; variants reduce protein levels and affect cell migration/apoptosis.
Yuan et al. (2024) [27]	*KDELR3*	Undetermined	ES	449 EM cases/9606 controls	Rare PTVs in *KDELR3* lead to scleral/choroidal thinning and elongated axial length.
Ye et al. (2024) [35]	*ARR3*, *P3H2*, *VPS13B*	AD, AR, XL	Trio-based ES	26 familial trios	Identified novel mutations in *ARR3* and *P3H2*; many cases linked to syndromic myopia genes.
Liu et al. (2025) [36]	*CHRNB1*	Undetermined	Sex-stratified ExWAS	15,582 individuals	*CHRNB1* deficiency specifically dysregulates mitochondrial organization in males, increasing HM risk.
Wang et al. (2025) [44]	*TCF7L2*, *AIPL1*, *INPP5E*, *SALL4*	De novo/AD/AR	ES	100 HM patients	Identified novel genes and de novo mutations (*ZEB1*, *HDAC8*); 56.52% mutation rate in eoHM.
Rui et al. (2025) [45]	*POLA1*, *HK1*, *GSN*, *COL5A1*, *CRYBB3*	AD, AR, XL	ES	47 unrelated families	Identified 7 ocular disease genes as potentially pathogenic for the first time in Chinese eoHM.

AD = autosomal dominant; AR = autosomal recessive; EM = extreme myopia; eoHM = early-onset high myopia; ES = exome sequencing; ExWAS = exome-wide association study; HM = high myopia; IRD = inherited retinal disease; LIAVA = p.V171I, p.I178V, and p.S180A but not p.L153M and p.A174V in *OPN1LW* haplotype; LIVVA = p.V171I, p.A174V, p.I178V, and p.S180A but not p.L153M in *OPN1LW* haplotype; LVAVA = p.I178V and p.S180A but not p.L153M, p.V171I, and p.A174V in *OPN1LW* haplotype; loHM = late-onset high myopia; PTV = protein truncating variant; RPE = retinal pigment epithelium; TES = targeted exome sequencing; XL = X-Linked.

## Data Availability

No new data were created or analyzed in this study. Data sharing is not applicable to this article.

## Abbreviations

The following abbreviations are used in this manuscript:

AD	Autosomal dominant
AOSW	Age-of-onset of spectacle wear
AR	Autosomal recessive
CNV	Copy number variation
CREAM	Consortium for Refractive Error and Myopia
CSNB	Congenital stationary night blindness
ECM	Extracellular matrix
EM	Extreme myopia
EoHM	Early-onset high myopia
ERG	Electroretinography
ES	Exome sequencing
ExWAS	Exome-wide association study
FAF	Fundus autofluorescence
FEVR	Familial exudative vitreoretinopathy
GS	Genome sequencing
GWAS	Genome-wide association study
GxE	Gene-environment
GxG	Gene-gene
HM	High myopia
IRD	Inherited retinal disease
LCA	Leber congenital amaurosis
LoHM	Late-onset high myopia
OCT	Optical coherence tomography
PRS	Polygenic risk score
PTV	Protein-truncating variant
RNFL	Retinal nerve fiber layer
RPE	Retinal pigment epithelium
SNP	Single nucleotide polymorphism
TES	Targeted exome sequencing
XL	X-linked
